# Different active exogenous carbons improve the yield and quality of roses by shaping different bacterial communities

**DOI:** 10.3389/fmicb.2025.1558322

**Published:** 2025-03-28

**Authors:** Shixiong Li, Yuanyang Peng, Manying Li, Xin Li, Haoyang Li, Xilatu Dabu, Yun Yang

**Affiliations:** ^1^College of Resources and Environment, Yunnan Agricultural University, Kunming, Yunnan, China; ^2^Yunnan Huayan Agricultural Science and Technology Co., Ltd., Kunming, China

**Keywords:** biochar, organic fertilizer, productivity, bacterial community, organic carbon fractions

## Abstract

The application of exogenous organic carbon represents a significant strategy for enhancing soil fertility and promoting sustainable agricultural development. This approach modifies the physicochemical properties of soil and influences microbial community structures, consequently improving crop yield and quality. Nevertheless, the mechanisms underlying microbial community responses to various forms of active exogenous organic carbon remain poorly understood and require further investigation. A 1-year follow-up experiment was conducted to examine the effects of different carbon sources on the yield and quality of cut roses, along with the characteristics of the soil bacterial community. The results indicated that applying organic fertiliser and biochar significantly enhanced the productivity of cut roses, demonstrating a sustained growth-promoting effect. Organic fertiliser provides more active, readily oxidisable organic carbon to the soil compared to biochar. In contrast, biochar supplies stable organic carbon, including inert organic carbon that is difficult to oxidise, firm organic carbon (FOC), and total inert organic carbon, which has a high degree of humification that significantly exceeds that of organic fertiliser. The application of biochar and organic fertiliser not only altered the abundance, diversity, and composition of the rhizosphere microbial community but also enriched beneficial microorganisms. Redundancy analysis results indicated that FOC, available phosphorus, and soil organic matter were the primary factors influencing the bacterial community. The results of this study demonstrated that exogenous organic carbon exerted positive and indirect effects on crop yield by influencing soil properties and bacterial communities. These findings provide novel evidence supporting the rational application of biochar and organic fertilisers as a means to promote agricultural sustainability in red soil regions.

## 1 Introduction

Intensive farming practices, combined with excessive water and fertiliser use, degrade critical soil functions, including the microbiome and soil organic carbon. These indicators are essential for maintaining soil ecosystem functions and ensuring agricultural productivity (Byers et al., 2024; Domeignoz-Horta et al., 2024). However, long-term over-application of mineral fertilisers can lead to environmental pollution and soil degradation, which further influences the health and processes of entire agroecosystems. Soil management practices in Southwest China are vital in this context, as it is an important agricultural production area in China (Li et al., 2023). Due to continuous planting and intensive cultivation, the soil in this area is gradually degrading (Hu et al., 2025). Moreover, because of the inherently low organic matter and low fertility characteristics of red soil, biodiversity in this area is low (Huang et al., 2020). Soil microorganisms play an important role in controlling plant growth and stress resistance and are often used as indicators of soil quality in assessments (Hartmann and Six, 2023; Yang et al., 2025). Therefore, to achieve the sustainable use of intensively cultivated red soil, it is necessary to select appropriate amendments to improve the physical, chemical, and biological properties of the soil.

The application of carbon-rich organic materials can enhance soil carbon activity and modify soil carbon fractions, thereby boosting microbial activity and crop productivity (Fu et al., 2024; Zhang et al., 2024). Organic fertilisers provide rich organic matter and essential nutrients that promote soil microbial activity and metabolism, maintaining or enhancing ecosystem services (Mishra et al., 2025; Song et al., 2022). In addition to organic fertilisers, biochar has been widely used as a soil conditioner or organic material in recent years for crop production and soil improvement. Its rich pore structure, nutrient content, and carbon (C) content can improve soil structure and nutrient supply while increasing carbon storage (Fan et al., 2024; Xiao et al., 2024). However, the effects of different organic materials on soil material cycles and carbon components vary (Liu et al., 2024). Soil microorganisms often exhibit substrate preferences and adopt distinct nutrient acquisition strategies that lead to their enrichment on specific substrates (Luo et al., 2023). However, the impact of organic materials on microbial diversity is inconsistent, and it remains unclear which microbial genera are most influenced by the activity of these materials. Changes in soil microbial communities caused by organic materials can further affect the functional traits of microorganisms, including their roles in maintaining soil nutrient cycling and crop productivity.

The quality of organic carbon sources is a key factor influencing soil microbial diversity (Shi et al., 2023). Easily available carbon sources, such as glucose, stimulate microbial growth and reproduction more effectively than cellulose (Peng et al., 2024). The exogenous addition of organic fertiliser and biochar is an effective measure to supplement soil carbon (Gross et al., 2021), and it was found that organic fertiliser could provide more active carbon sources for the soil than biochar, whereas biochar is mainly rich in stable carbon sources (Hu L. et al., 2024). However, different decomposition methods yield varied results in determining organic carbon. These organic carbons cannot be completely oxidised by different oxidants due to their unique chemical structures, which affect nutrient adsorption.

Due to their unique chemical structures, energy, and efficiency available to microorganisms, the utilisation efficiency of microbial carbon is also significantly different, which is bound to affect the diversity of soil microorganisms (Chen and Hu, 2024). Biochar improves crop growth by regulating substrate porosity and nutrient availability, as well as enhancing the soil microenvironment through microbial group regulation, thereby increasing crop productivity (Agegnehu et al., 2017; Wang et al., 2023). Organic fertilisers promote plant growth by recruiting beneficial endophytes, which further enhance crop productivity (Pariona-Llanos et al., 2010). These fertilisers have shown considerable benefits in regulating soil properties and promoting plant growth in degraded soils (Song et al., 2022). Extensive research indicates that applying organic fertilisers can enhance soil organic carbon content, improve soil structure, increase cation exchange capacity, and boost nutrient availability (Tian S. et al., 2022). Additionally, organic fertilisers can stimulate soil microbial biomass and induce changes in community structure and abundance (Liu et al., 2022). However, it is crucial to note that applying organic fertilisers does not necessarily enhance soil microbial diversity; their effects depend on factors such as the duration of application, the source and nature of the fertiliser, soil type, and farming conditions (Li et al., 2023; Mishra et al., 2025). Therefore, to achieve sustainable development in long-term intensive agricultural production systems, further investigation into the impacts of organic fertilisers on soil physicochemical properties and microbial communities is essential. For perennial crops, understanding the relationship between soil carbon components, the microbiome, and plant productivity under long-term intensive cultivation is particularly significant. Additionally, the composition of the soil carbon components must be defined scientifically.

Cut roses (Rosa cvs.) belong to the Rosaceae family in plant taxonomy. Rosa cvs is a cash crop widely cultivated in southwest China, covering an area of approximately 1.94 million mu (Ma et al., 2022). It is one of the world’s three major flower-producing regions. However, long-term, large-scale planting has caused significant soil degradation in the region, which is not conducive to sustainable development (Burian et al., 2024). Previous studies have found that the use of organic fertilisers and biochar can improve soil quality and plant performance. Research has also indicated that rhizosphere bacteria can directly promote plant growth. However, the effects of organic fertilisers and biochar amendments on the rhizosphere microbial communities of cut roses in red soil have not been thoroughly investigated. Therefore, this study aims to examine the effects of organic fertiliser and biochar application on soil nutrient profiles and physicochemical properties, rhizosphere bacteria, yield, and quality of cut roses in red soil that has undergone long-term intensive cultivation. The study discovered that the application of various active carbon sources can improve soil organic carbon components, microbial community composition, and soil functions (such as nutrient retention and availability), thereby increasing the productivity (yield and quality) of cut roses.

## 2 Materials and methods

### 2.1 Study site and materials

The experiment was conducted from June 2023 to June 2024 at the Flower Soil Monitoring Station of Yunnan Agricultural University (102°36′N, 24°42′E) in Jinning District, Kunming City, Yunnan Province. The tested soil had the following basic properties: pH 5.4; organic matter 12.91 g/kg; alkali-hydrolysable nitrogen 57.4 mg/kg; available phosphorus 16.54 mg/kg; and available potassium 60.30 g/kg.

#### 2.1.1 Tested biochar

The biochar used in this study was derived from rubber wood, carbonised under anoxic conditions at 450°C, with a pH of 8.7, containing N 0.11%, P_2_O_5_ 0.02, and 37.92% organic carbon. It is sourced from Xishuangbanna, Yunnan Province.

#### 2.1.2 Tested organic fertiliser

Kunming Lishan Technology Development Co., Ltd. provided a commercial organic fertiliser containing Chinese medicine residue and tobacco. It has a pH of 7.6, N of 1.1%, P_2_O_5_ of 1.61%, K_2_O of 3.92%, and organic carbon of 21.85%.

The experiment used balanced fertiliser (20–20–20), high-potassium fertiliser (9–12–40), and high-phosphorus fertiliser (10–52–10).

The test crop was the multi-headed cut rose variety “Miss Nana.”

### 2.2 Test design

The experiment set up one control and three treatments: CK (single application of chemical fertiliser), B (45,000 kg/hm^2^ biochar), OF (45,000 kg/hm^2^ organic fertiliser), and BF (22,500 kg/hm^2^ organic fertiliser +22,500 kg/hm^2^ biochar, carbon-based organic). The application rate of biochar and organic fertiliser, set at 45,000 kg/hm^2^, was determined through previous research and practical verification (Li et al., 2025; Yang et al., 2023). Since the application of biochar and organic fertiliser introduces additional nutrients, the nutrient contributions from biochar and organic fertiliser were calculated separately while ensuring equivalent total nutrient inputs. For the Control (CK) treatment, deficient nutrients were supplemented with urea, calcium superphosphate, and potassium chloride to achieve equivalent amounts of nitrogen (N), phosphorus (P), and potassium (K). Biochar and organic fertiliser were applied as basal fertilisers in a single application before transplanting. The control (CK) treatment also received equivalent nutrient amounts through the application of urea, calcium superphosphate, and potassium chloride. Organic materials were mixed with soil in a 60 cm soil layer using rotary tillage to accumulate moisture at that depth. Drip irrigation and fertilisation were performed at the test sites. Before each fertilisation, drip irrigation was performed for 25 min, followed by the initiation of fertiliser injection and an additional 30 min of drip irrigation. The irrigation amount applied to each plot remained consistent. Measures for pest and disease prevention, as well as weeding, were carried out simultaneously.

The specific fertilisation measures during the experiment were as follows: for the first crop (from June 22, 2023, to December 22, 2023), a balanced fertiliser was applied for 7 days, followed by high-potassium and high-phosphorus fertilisers for 2 months; each treatment’s fertiliser application rate was consistent. The total amount of fertiliser applied was N 178.51 kg/hm^2^, P_2_O_5_ 220.71 kg/hm^2^, and K_2_O 207.59 kg/hm^2^. For the second crop (from December 23, 2023, to June 23, 2024), balanced fertiliser was also applied for 7 days, with high-potassium and high-phosphorus fertilisers applied for 2 months, resulting in a total amount of N 187.88 kg/hm^2^, P_2_O_5_ 231.96 kg/hm^2^, K_2_O 245.09 kg/hm^2^. The total number of branches harvested from each plot during the harvest period was calculated as the yield. The first crop was collected three times to determine the average yield, A-level flower rate, and output value, while the second crop was measured for the first crop. Quality grading of cut roses followed the local standard DB 53/T 105–2003 ‘fresh cut flower quality grade’ in Yunnan Province, ranking from best to worst as A, B, and C.

### 2.3 Sample collection

On June 23, 2024, soil samples were collected, uniformly sifted through a 2-mm sieve, and placed into sterile bags. The samples were divided into two parts: one part was dried and stored at room temperature to test soil carbon components along with five routine physicochemical parameters—soil organic matter (SOM), alkali-hydrolysable nitrogen (AN), available phosphorus (AP), available potassium (AK), and pH. The other part of the soil samples was packed in sterile tubes, transported back to the laboratory, and immediately stored in an ultralow-temperature freezer at −80°C to determine the diversity of soil bacterial community structure.

### 2.4 Sample determination

Total organic carbon (TOC) was measured using a TOC analyser (Jena multi N/C 2100 TOC total organic carbon/total nitrogen analyser, Germany). Soil organic carbon (SOC) was determined using the potassium dichromate oxidation-colorimetric method. Under external heating conditions (oil bath temperature of 170°C–180°C, boiling for 5 min), soil organic matter (carbon) was oxidised with a specific K_2_Cr_2_O_7_-H_2_SO_4_ solution, and the remaining potassium dichromate was titrated with ferrous sulphate. The content of organic carbon was calculated from the amount of K_2_Cr_2_O_7_ consumed. The specific extraction process of SOC follows the method described by Chen et al. (2022). Readily oxidisable organic carbon (ROC) was determined using the 333 mmol/L potassium permanganate oxidation-colorimetric method. The organic carbon in the soil that can be oxidised by the 333 mmol/L KMnO4 solution is defined as easily oxidised organic carbon. According to the difference between the concentration of potassium permanganate (residual liquid) used to oxidize the easily oxidised carbon in the soil sample and the control concentration (theoretically 333 mmol/L), the molar mass of potassium permanganate consumed by easily oxidised carbon in various soil samples is quantified, and then the content of easily oxidised organic carbon in each soil is calculated. The specific determination method of SOC follows the method of Li et al. (2025). Five routine soil parameters were determined based on Boschdan’s ‘soil agrochemical analysis’ (Jiang et al., 2025).

### 2.5 Determination of soil microbial diversity

Total soil DNA was extracted according to the instructions provided by the DNeasy Power Soil Kit (QIAGEN), and the mass concentration and purity of the extracted DNA were determined using a Novaseq 6000 PE250 platform. The V4-V5 region of the bacterial target, the 16S rRNA gene, was amplified. The purity and concentration of the extracted DNA were assessed through agarose gel electrophoresis. An appropriate amount of DNA was then placed in a centrifuge tube and diluted with sterile water to reach a concentration of 1 ng/μl. Using the diluted genomic DNA as a template, specific primers with barcodes, along with New England Biolabs’ Phusion^®^ High-Fidelity PCR Master Mix with GC Buffer and a high-efficiency high-fidelity enzyme, were selected for PCR based on the amplification region. To ensure amplification efficiency and accuracy, PCR products were examined using a 2% agarose gel electrophoresis, and sample amounts were combined according to the concentration of the PCR products. After thorough mixing, the samples were re-evaluated using a 2% agarose gel electrophoresis. The target bands were recovered using the gel recovery kit provided by Qiagen. The NEBNext^®^ Ultra^™^ II DNA Library Prep Kit was used to construct the library, which was then quantified using Qubit and Q-PCR. Once the library was validated, the NovaSeq 6000 was used for on-machine sequencing.

According to the barcode sequence and PCR amplification primer sequence, each sample dataset was separated from the disembarkation data. After the removal of barcode and primer sequences using FLASH (V1.2.11)^1^ (Magoč and Flash, 2011), the reads from the samples were processed to generate raw tags. Then, the fastp software was used for control of the raw tags, resulting in high-quality clean tags. Finally, Usearch software was used to compare clean tags with the database to detect chimaeras and remove them (Haas et al., 2011) so as to obtain the final effective data, that is, effective tags. For the above effective tags, the DADA2 module in QIIME2 software was used to filter out sequences with an abundance of less than five (Callahan et al., 2016), yielding the final amplicon sequence variants (ASVs) and the feature list. The resulting ASVs were then compared to the database using the Classify-Sklearn module in QIIME2 (Bokulich et al., 2018; Bolyen et al., 2019) to retrieve species information for each ASV.

#### 2.5.1 Alpha diversity

QIIME2 software is used to calculate observed_otus, Shannon, Simpson, and Chao1 indices to assess the species diversity differences (Segata et al., 2011; Warton et al., 2012).

#### 2.5.2 Beta diversity

First, the UniFrac distance is calculated using QIIME2 software, and the dimensionality reduction graphs for NMDS are drawn using R software (version 4.3.2).^2^ Then, the adonis and anosim functions in QIIME2 software were used to analyse the significant differences in community structure between groups. Finally, species analysis highlighting significant differences between groups was performed using LEfSe or R software. The LEfSe analysis is performed using LEfSe software, with a default LDA score threshold of 4. Using R software, a MetaStat analysis was conducted to test the differences between the two comparison groups across six classification levels: phyla, class, order, family, genus, and species, yielding *p*-values. Species with a *p*-value less than 0.05 were identified as significantly different between groups. The T-test also uses R software to analyse significant differences in species at each taxonomic level (Kierepka and Latch, 2015) (The specific sequencing was performed by Tianjin Jizhi Gene Biotechnology Co., Ltd.).

### 2.6 Calculation parameters

TOC is the organic carbon determined by the combustion method at approximately 1,300°C by the TOC instrument, which can oxidise all organic matter, including aromatic hydrocarbons and other organic matter (Hou et al., 2024; Yan et al., 2024). Consequently, we define IOC_S_ as refractory organic carbons composed mainly of alcohols, ethers, aldehydes, ketones, and carboxylic acids. IOC_T_ is described as inert organic carbon mainly composed of alcohols, ethers, aldehydes, ketones, carboxylic acids, and aromatic hydrocarbons. Firm organic carbon (FOC) is defined as stable organic carbon chiefly consisting of aromatic hydrocarbons, pyridine, and volatile linear aliphatic compounds.

SOC components-related calculation formula:

${IOC}_{S}=SOC-\mathrm{ROC}$

${IOC}_{T}=TOC-\mathrm{ROC}$

FOC=TOC−SOC

Because the decomposition methods of soil carbon components in Section 2.4 are different, they have a great influence on the properties of organic carbon; therefore, the IOC_S_ in this study represent organic carbon that is difficult to oxidise, IOC_T_ represents total inert organic carbon, and FOC represents firm organic carbon. Therefore, this study indicates that the organic carbon components with different activities are ROC, IOC_S_, IOC_T_, and FOC, and their stability is enhanced.

Calculation formula of humification rate:

$\begin{array}{l} \mathrm{Humification rate}\;\left(\%\right)=(\mathrm{soil organic carbon content after}\\ 1\;\mathrm{year}-\mathrm{soil background value})/\mathrm{organic carbon input}\text{.} \end{array}$

Grade A flower rate calculation formula:

$\begin{array}{l} \mathrm{Grade}\;A\;\mathrm{flower rate}\;\left(\%\right)=\mathrm{Grade}\;A\;\mathrm{flower branch number}\\ \mathrm{per}\;\mathrm{treatment}/\mathrm{total branch number}\;\mathrm{per}\;\mathrm{treatment}\text{.} \end{array}$

Yield calculation formula:

$\begin{array}{l} \mathrm{Yield}\;\left(\mathrm{branch}/{\mathrm{hm}}^{2}\right)=\mathrm{total number of}\;\\ \mathrm{branches harvested}\;\mathrm{per}\;\mathrm{plot}/12\times 10,000. \end{array}$

Output value calculation formula:

$\begin{array}{l} \mathrm{Output value}=A-\mathrm{grade flower number}\times A\\ -\mathrm{grade flower unit price}+B-\mathrm{grade flower number}\\ \times \;B-\mathrm{grade flower unit price}+C-\mathrm{grade flower number}\\ \times \;C-\mathrm{grade flower unit price}\text{.} \end{array}$

### 2.7 Data analysis

Excel 2019 software was used to process the data, SPSS 26.0 was used for variance analysis, Canoco 5 was used for redundancy analysis, Origin 2021 software was used for plotting, and the Duncan method was used to test the significance of the differences among the treatments. Spearman correlation analysis was conducted to analyse the relationship between soil physical and chemical properties and the bacterial genus level.

## 3 Results

### 3.1 Effects of biochar and organic fertiliser application on yield and quality of cut rose

As shown in Table 1, compared to the control (CK), the organic fertiliser (OF), biochar (B), and carbon-based organic (BF) treatments significantly increased the yield, A-class flower rate, and output value of the first-cut rose. The yield increased by 31.25, 19.79, and 52.08%, while the A-class flower rate increased by 25.60, 15.38, and 33.08%, respectively. Among these, the OF treatment exhibited better results than the B treatment, and the BF treatment had the greatest effect on increasing the yield and A-class flower rate. Compared to the CK, the OF, B, and BF treatments enhanced the yield and output values of cut roses. While the OF treatment was superior to the B treatment, the difference was not statistically significant. However, the OF treatment significantly improved the A-grade flower rate by 57.78, 73.33, and 80.00%, respectively. The OF treatment was more effective than the B treatment, with the BF treatment yielding the best results. In contrast to the single application of chemical fertiliser, the application of biochar and organic fertiliser can significantly boost the productivity of cut roses.

**Table 1:** Effects of biochar and organic fertiliser on yield and quality of cut rose.

Treatment	The first crop			The second crop		
	Yield (ten thousand/hm^2^)	Grade A flower rate	Output value (ten thousand/hm^2^)	Yield (ten thousand/hm^2^)	Grade A flower rate	Output value (ten thousand/hm^2^)
CK	0.96 ± 0.079d	0.49 ± 0.12c	2.30 ± 0.47d	1.77 ± 0.50a	0.45 ± 0.17b	5.10 ± 1.46b
OF	1.26 ± 0.057b	0.73 ± 0.21a	4.31 ± 0.19b	2.67 ± 0.76a	0.71 ± 0.07a	9.01 ± 2.57a
B	1.15 ± 0.04c	0.68 ± 0.20b	3.77 ± 0.12c	2.44 ± 0.70a	0.78 ± 0.04a	8.80 ± 2.52a
BF	1.46 ± 0.08a	0.78 ± 0.19a	4.96 ± 0.26a	2.78 ± 0.80a	0.81 ± 0.06a	9.49 ± 2.71a

Different lowercase letters indicate that there are significant differences between different treatments (*p* < 0.05).

### 3.2 Effects of biochar and OF on soil carbon fractions

**Figure 1:**
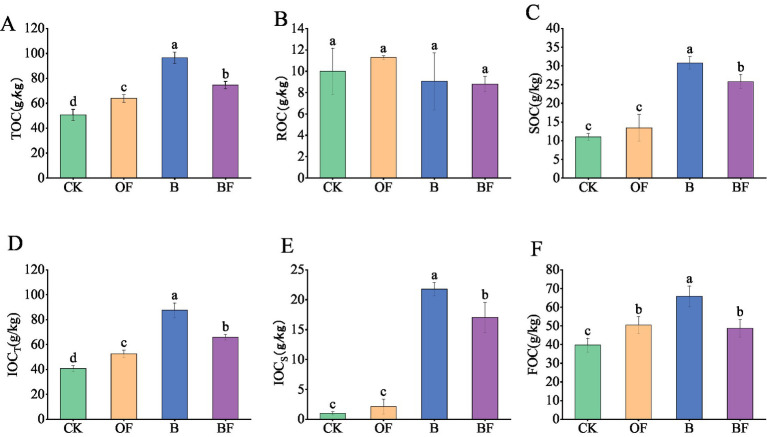
Effects of biochar and organic fertiliser on soil carbon fractions. **(A)** Total organic carbon content; **(B)** readily oxidizes organic carbon content; **(C)** soil organic carbon content; **(D)** total inert organic carbon content; **(E)** organic carbon that is difficult to oxidise content; **(F)** firm organic carbon content. Different letters indicate significant differences (Tukey’s HSD test, *p* < 0.05).

The soil carbon components were sampled and analysed after 1 year of experimental treatment (Figure 1). The contents of TOC and soil organic carbon (SOC) in treatment B were significantly higher than those in the OF treatment (Figures 1A,B). The content of active organic carbon (ROC) in the OF treatment was significantly higher than that in treatment B (Figure 1C). The stable organic carbon content in treatment B was significantly higher than that in treatment OF, regardless of the decomposition method. The contents of active and stable organic carbon in the BF treatment were intermediate between those in the OF and B treatments (Figures 1D,E). For stable organic carbon (FOC), treatment B was significantly higher than treatment OF (Figure 1F). The application of OF and biochar has different effects on soil carbon components. OFs can provide a more active source of carbon for the soil than biochar, whereas biochar is rich in stable inert carbon.

### 3.3 Effects of biochar and organic fertiliser on soil chemical properties

The impact of organic fertiliser and biochar application on the soil properties and nutrient profile of red soil is shown in Table 2. In general, biochar amendment and organic fertiliser caused significant changes in most soil parameters (*p* < 0.05). Compared to CK, the OF, B, and BF treatments significantly increased soil organic matter (SOM) by 22.08, 180.54, and 135.03%, respectively. The amount of alkali-hydrolysable nitrogen significantly increased by 34.23, 95.56, and 86.71%, respectively. The available phosphorus content in the OF treatment significantly increased by 31.28%, whereas that in the B and BF treatments significantly decreased by 17.28 and 16.77%, respectively. Available potassium increased significantly by 55.70, 21.74, and 72.48%, respectively. The pH values increased by 0.11, 0.23, and 0.17 units, respectively. Overall, the input of biochar and OF enhanced the degree of soil humification, with the order being B treatment > BF treatment > F treatment, where the B treatment exhibited the highest degree of humification, showing similar humus stability (Supplementary Figure A1). The application of biochar, organic fertiliser, and their combination will significantly affect soil chemical properties and soil humification.

**Table 2:** Effects of biochar and organic fertiliser application on soil chemical properties.

Treatment	SOM (g/kg)	AN (mg/kg)	AP (mg/kg)	AK (mg/kg)	pH	Humification rate (%)
CK	18.93 ± 1.59c	139.07 ± 9.83d	26.06 ± 1.97c	43.64 ± 5.68c	5.92 ± 0.09c	–
OF	23.11 ± 3.31c	186.67 ± 15.42c	34.22 ± 0.75a	67.94 ± 3.96a	6.03 ± 0.03b	1.38 ± 0.45c
B	53.11 ± 2.91a	281.96 ± 1.75a	28.53 ± 1.72b	53.12 ± 1.26b	6.15 ± 0.03a	4.10 ± 0.30a
BF	44.50 ± 3.22	259.65 ± 3.12b	35.10 ± 3.73a	75.26 ± 3.54a	6.09 ± 0.03ab	3.68 ± 0.37b

SOM, soil organic matter content; AN, alkali-hydrolysable nitrogen content; AP, available phosphorus content; AK, available potassium content. After the same column of data, different lowercase letters indicate significant differences between treatments (*p* < 0.05).

### 3.4 Effects of biochar and OF on soil microbial composition

**Figure 2:**
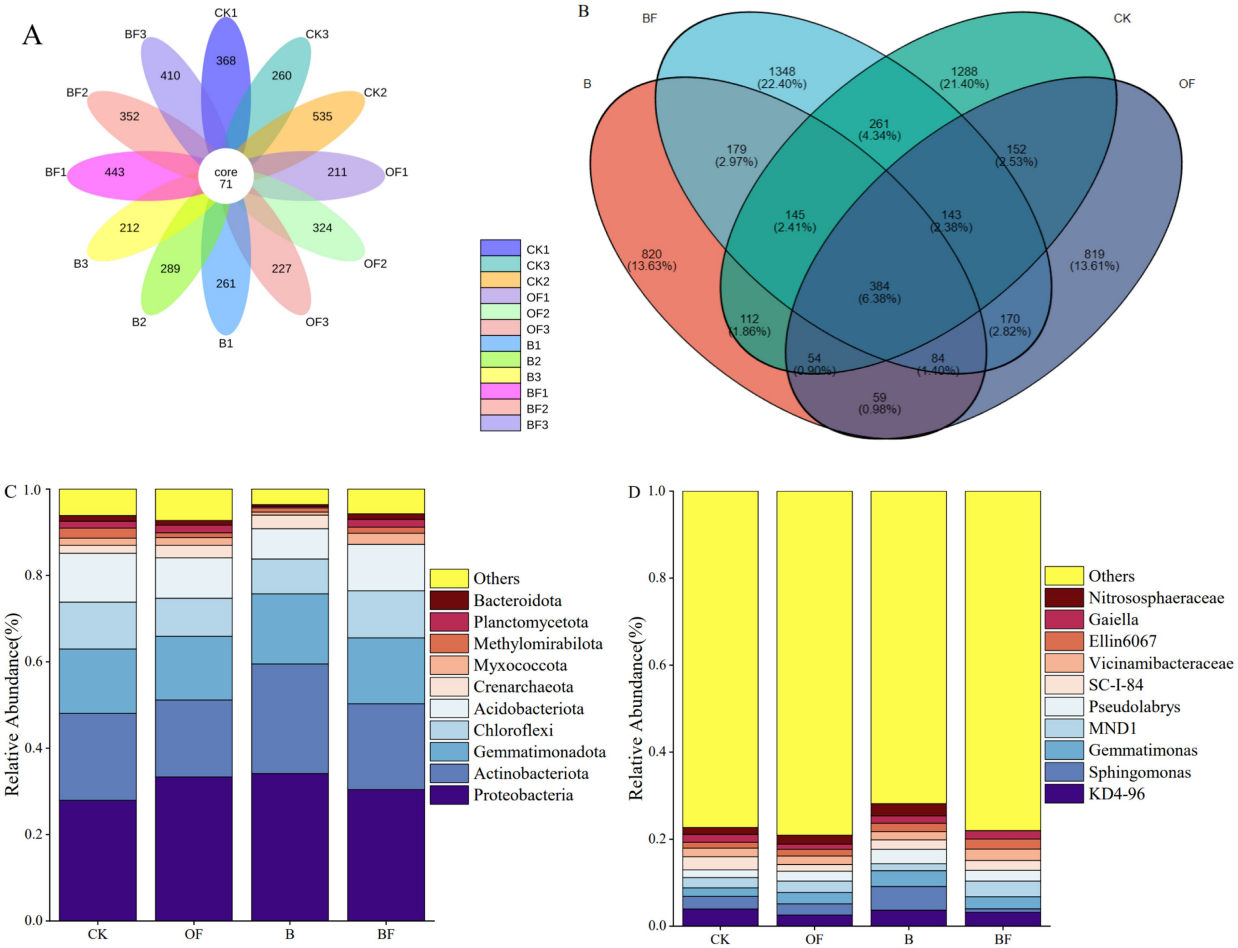
Venn diagram analysis of microorganisms under different treatments and abundance analysis at different levels. **(A)** Petal diagram analysis under different treatments. **(B)** Venn diagram analysis under different treatments. **(C)** Comparison of the horizontal abundance of species in different treatments. **(D)** Comparison of different treatments at the genus level.

The bacterial diversity in the cut rose soil samples was analysed via their biological replicates. Petal graph analysis shows that (Figures 2A,B), each circle represents a sample (group), the number of overlapping parts of the circle and the circle represents the number of ASVs shared between the samples (groups), and the number of non-overlapping parts represents the number of unique ASVs of the sample (group). The number of ASVs shared by all treatments was 71. The Venn diagram showed that in BF and B treatments, BF treatment contained 2714 ASVs, and B treatment contained 2891 ASVs; among them, 1922 ASVs were unique to BF treatment and 1045 were unique to B treatment. In BF and CK treatments, CK contained 2539 ASVs, of which 1606 ASVs were unique to CK; in BF and OF treatments, OF treatment contained 1865 ASVs, of which 1132 ASVs were unique to OF treatment. There were 733 ASVs in CK and OF treatments, 695 ASVs in CK and B treatments, 933 ASVs in CK and BF treatments, 581 ASVs in OF and B treatments, 781 ASVs in OF and BF treatments, and 792 ASVs in B and BF treatments. The relative abundances of soil bacteria after the application of biochar and OF were compared at the phylum and genus levels. Differences in community composition were observed. As shown in Figure 2C, each treatment mainly comprised Proteobacteria, Actinobacteria, Gemmatimonadetes, Chloroflexi, and Acidobacteria. Figure 2D shows the relative abundance of microbial genera under the different treatments; the abundance of each treatment was relatively low. *KD4-96*, *Sphingomonas*, *Gemmatimonas*, *MND1*, *Pseudolabrys*, *SC-I-84*, *Vicinamibacteraceae*, *Ellin6067*, *Gaiella*, and *Nitrososphaeraceae* were the top 10 species; however, the abundance of microorganisms among the treatments also differed. For example, a homogeneity of variance test was performed using Levene’s Test method, and then a one-way analysis of variance was performed at a significance level of *p* < 0.05. *Sphingomonas* and *Pseudolabrys* were significantly enriched in treatment B, which was significantly higher than in the other treatments. In summary, the bacterial community compositions of the different treatments differed significantly. The enrichment of beneficial bacteria such as *Sphingomonas* and *Pseudolabrys* in the soil may be the key factor leading to an increase in the A-grade flowering rate of cut roses. The abundance of *Sphingomonas* and *Gemmatimonas* was higher in the OF treatment, indicating that these microorganisms play an active role in maintaining the soil health of cut roses. These analyses enhance understanding of dynamic changes in soil microbial ecology and provide a microbiological basis for soil quality control in cut rose cultivation.

### 3.5 Microbial diversity index analysis

**Figure 3:**
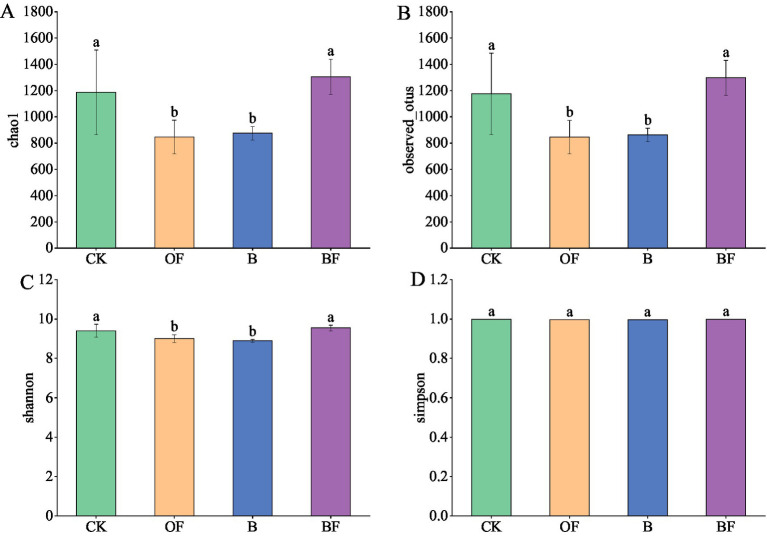
Bacterial *α* diversity index. Chao1: **(A)** Estimate the total number of species contained in the community sample; the more low-abundance species in the community, the greater the Chao1 index; **(B)** observed _ OTUS: the number of species observed intuitively; the larger the index, the more species observed; **(C)** the higher the community diversity, the more uniform the species distribution, the greater the Shannon index; **(D)** Simpson: Characterise the diversity and evenness of species distribution in the community. The better the species evenness, the greater the Simpson index.

The application of biochar and OF significantly changed the richness and diversity of the soil bacteria (Figure 3). The Chao1, observed OTUs, Shannon, and Simpson indices of the soil samples were compared. The results showed that both F and B treatments reduced the Chao1, observed OTUs, Shannon, and Simpson indices. In contrast, the BF treatment increased the Chao1, observed OTUs, Shannon, and Simpson indices, which are beneficial for microbial survival. The Chao1 index of the CK treatment was high, with a greater number of low-abundance species. This indicates that the application of biochar and OFs reduces bacterial diversity during intensive facility cultivation.

### 3.6 Analysis of soil differential microorganisms under biochar and OF application

**Figure 4:**
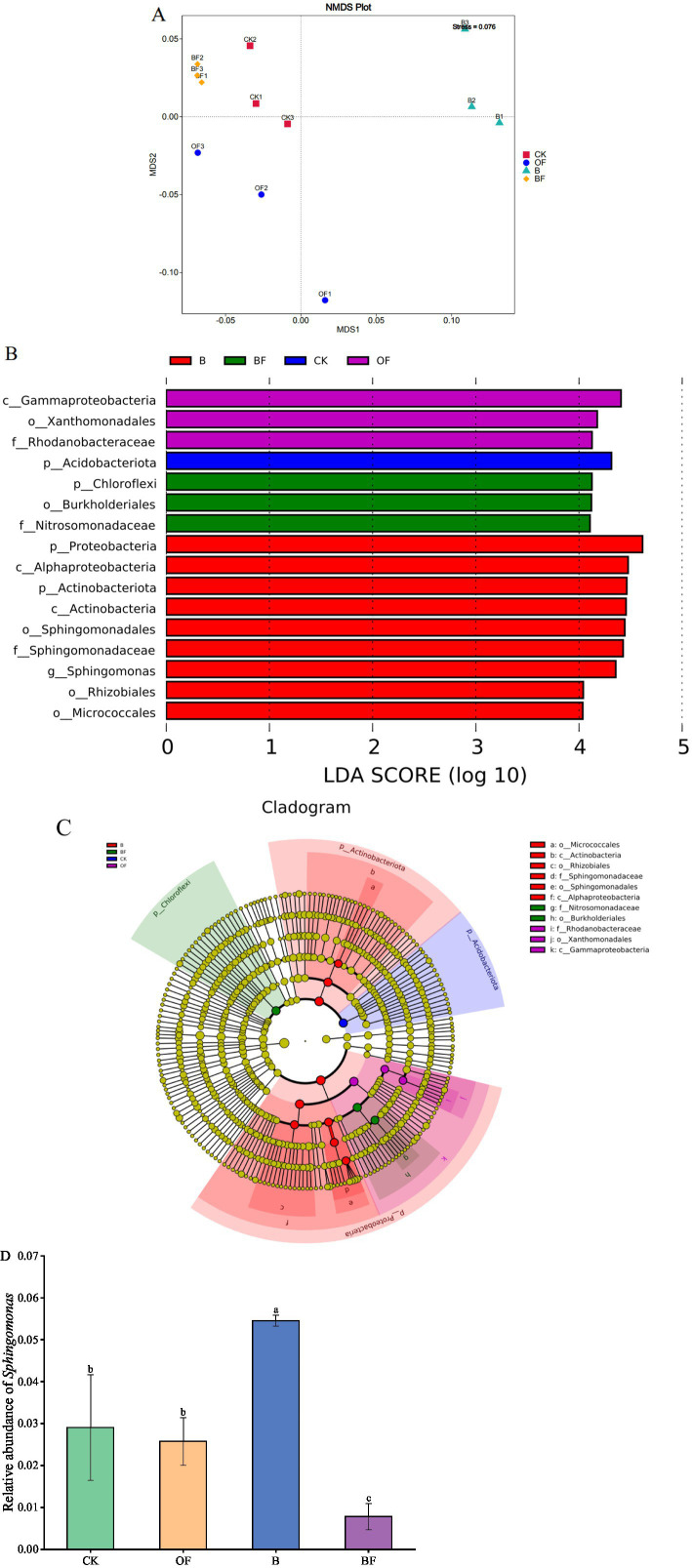
Effects of biochar and organic fertiliser on bacterial community composition. **(A)** NMDS analysis, where different colours or shapes represent samples from different populations. The closer the two sample points are, the more similar their species composition is. The abscissa and ordinate represent the relative distance, which is of no practical significance. Stress: Test the quality of NMDS analysis results. When the general stress is less than 0.2, the NMDS two-dimensional diagram can be used, and the diagram has certain explanatory value. When the stress is less than 0.1, it is considered that the sorting is better. The stress value in the diagram is represented by two decimal places. **(B)** The significance test of the difference between the groups at the bacterial genus level. **(C)** In the evolutionary branch diagram, the circles radiated from the inside to the outside represent the classification level from the phylum to the genus (or species). Each small circle at different classification levels represents a classification at this level, and the diameter of the small circle is proportional to the relative abundance. Colouring principle: The species with no significant difference were uniformly coloured as yellow, and the Biomarker of the different species followed the group for colouring. The red node represents the microbial group that plays an important role in the red group, and the green node represents the microbial group that plays an important role in the green group. If a group is missing in the figure, it indicates that there is no significant difference in this group, so this group is missing. The species names represented by the English letters in the figure are displayed in the right legend. **(D)** *Sphingomonas* The relative abundance of genera in different treatments. Different letters indicate significant differences (Tukey’s HSD test, *p* < 0.05).

Based on the Bray–Curtis distance algorithm, NMDS analysis was used to compare differences in soil bacterial community composition after the application of biochar and OF (Figure 4). The composition of the soil bacterial community was analysed at the genus level (Figure 4A). The results indicated a significant difference in soil bacterial community composition after the application of biochar and OF (stress = 0.0076). Linear discriminant analysis (LDA) effect size [LEfSe] analysis, with an LDA threshold of 4 (*p* < 0.05), was used to identify dominant microorganisms in the soil (Figures 4B,C). The results showed that, at the genus level, *Sphingomonas* was significantly enriched in treatment B and had a significant effect on this difference, indicating that *Sphingomonas* may play a key role in the growth of cut roses (Figure 4D).

### 3.7 The relationship between soil carbon fractions, physical and chemical properties, and soil microorganisms

**Figure 5:**
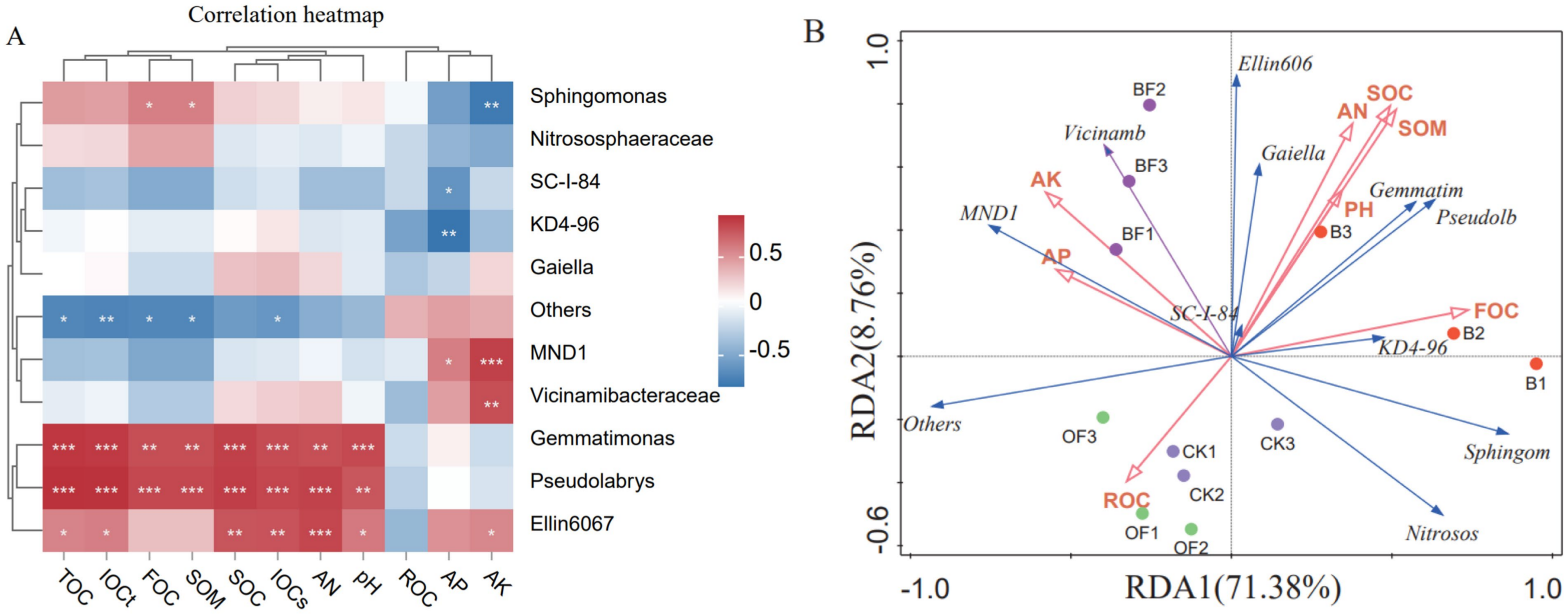
Correlation analysis and RDA analysis of soil carbon components, physical and chemical properties and bacterial community. **(A)** Correlation heat maps of soil carbon composition, physical and chemical properties, and bacterial communities. **(B)** Redundancy analysis of soil carbon components, physical and chemical properties and bacterial communities. TOC, total organic carbon; SOC, soil organic carbon; IOC_S_, organic carbon that is difficult to oxidise; IOC_T_, Total inert organic carbon; FOC, Firm organic carbon; AK, available potassium; AN, available nitrogen; SOM, soil organic matter; ROC, readily oxidizes organic carbon; *p* > = 0.05 unmarked, 0.01 < *p* < 0.05 marked: *, 0.001 < *p* < 0.01 marked: **, *p* < = 0.001 marked: ***.

Spearman correlation analysis was conducted between the relative abundances of the top 10 bacterial genera and soil carbon components, along with physical and chemical properties. At the genus level, *Sphingomonas* was positively correlated with FOC and SOM and negatively correlated with AK, while *Gemmatimonas* and *Pseudolabrys* were significantly positively correlated with IOCT, IOCS, TOC, SOC, FOC, AN, and pH (Figure 5A). Figure 5B shows the correlation between eight physicochemical properties and 10 key bacterial genera at the genus level. The first two axes of redundancy analysis (RDA1 and RDA2) explained 80.14% of the variation related to environmental factors (with RDA1 accounting for 71.38% and RDA2 for 8.76%). The correlation between species data and environmental factors reached 0.9488 and 0.9806 for RDA1 and RDA2, respectively (verified by a permutation test, *p* < 0.05), indicating that the ordination results reliably reflect the relationship between bacterial communities and environmental factors. FOC, AP, and SOM explained 40.30% (*p* < 0.01), 29.60% (*p* < 0.01), and 8.20% (*p* < 0.05) of the variation in bacterial community composition, respectively, making them the main environmental factors affecting the bacterial community.

## 4 Discussion

### 4.1 Effects of biochar and OF on soil carbon fractions

The application of exogenous carbon significantly alters soil microorganisms and affects soil carbon components (Das et al., 2023). This study demonstrated that the input of biochar and organic fertiliser significantly changed the soil carbon components and increased the contents of soil TOC, SOC, ROC, organic carbon that is difficult to oxidise (IOC_S_), total inert organic carbon (IOC_T_), and firm organic carbon (FOC). OFs can input more active carbon sources for soil than biochar (Hu L. et al., 2024). In this study, the input of OF was more likely to input ROC for soil. The effects of biochar on SOC, IOC_S_, IOC_T_, and FOC were better than those of the OF. This is primarily because biochar is rich in cellulose and lignin carbons and forms aromatic hydrocarbons during pyrolysis, which are not easily utilised by microorganisms. OFs mainly contain active carbon sources, such as glucose, and their carbon bioavailability is higher than that of biochar. Therefore, the effect of improving ROC was greater than that of biochar, and the effect of biochar on improving the stable carbon content was greater than that of OF (Plaza et al., 2016). The results of the organic carbon determination by different decomposition methods also differed. It is generally believed that potassium dichromate can oxidise most organic compounds but cannot completely oxidise aromatic hydrocarbons, pyridine, volatile linear aliphatic compounds, and other organic compounds (Knicker et al., 2007; Quan et al., 2017). Potassium permanganate (333 mmol/L) can oxidise amino acids and simple sugars but cannot oxidise alcohols, ethers, aldehydes, ketones, carboxylic acids and their derivatives, and aromatic hydrocarbons (Zhang et al., 2005). These different organic carbons are bound to affect soil microbial communities because of their different chemical structures and microbial utilisation efficiencies (Peng et al., 2024). It may have recruitment, activation, and other effects (Tian X. et al., 2022; Wang et al., 2021). The effect of biochar on stable carbon was better than that of OFs. This may be due to the formation of various organic compounds during the pyrolysis of biochar, which leads to the improvement of biochar to improve stable organic carbon due to the OF. Therefore, we found that the input of biochar provided a more stable carbon source than OF, whereas the input of OF provided a better active carbon source than biochar and significantly changed the soil carbon composition. The effects of exogenous organic carbon sources on soil carbon fractions, nutrient availability, and soil ecosystem multifunctionality were examined.

### 4.2 Effects of biochar and OF on yield and quality of cut rose

Crop yield and quality are affected by the cultivation conditions, crops, and other factors. Soil nutrient status and microecology are important factors affecting crop yield and quality (Zhao et al., 2022). The yield and grade A flower rates are important indices for evaluating the quality of cut roses. In this study, biochar, OF, and their combined application significantly increased the yield and quality of cut roses. The effect of the first OF was better than that of biochar, and the effect of the second biochar was better than that of the OF; however, the difference was not significant. The combined application of the two was the best in terms of increasing the yield and A-class flower rate (Chen et al., 2022). The yield and quality of pitaya were also confirmed by the application of biochar and organic fertiliser. Previous studies have shown that the A-level rate of cut roses is generally approximately 20–40% (Zen et al., 2021); therefore, biochar, organic fertiliser, and their combined application significantly increased the yield of cut roses, the A-grade flowering rate, and improved crop productivity.

### 4.3 Effects of biochar and organic fertiliser on soil physical and chemical properties

Adding appropriate soil amendments and adopting appropriate fertilisation methods can effectively improve the soil nutrient status, crop yield, and quality (Bai et al., 2022; Li et al., 2021). In this study, the application of biochar and organic fertiliser significantly increased the amount of SOM, alkali-hydrolysable nitrogen, available phosphorus, available potassium, and pH. Previous studies have shown that when organic additives are used to modify the soil, it will affect the nutrient availability of the soil (Zhang et al., 2021).

After the application of biochar and organic fertiliser, the levels of soil organic matter, available nitrogen, available phosphorus, and available potassium increased significantly, and nutrient availability improved, partly aligning with the conclusion of Li et al. (2025). This observed phenomenon may be attributed to the porous structure of biochar, which allows for the attachment of abundant functional groups, thereby enhancing its capacity for nutrient adsorption in the soil. Concurrently, the porous architecture provides habitats for microorganisms, notably enriching the genus *Sphingomonas* (Figure 4D). This microbial enrichment subsequently accelerates the activation of soil nutrients through biochemical processes (Jiang et al., 2023).

The increased nutrient availability following organic fertiliser application could be explained by its high content of labile carbon, which promotes the formation of soil aggregates and consequently improves nutrient accessibility. Both biochar and organic fertilisers induce modifications in soil environmental parameters, including nitrogen, potassium, and phosphorus levels, as well as pH values (Li et al., 2023; Li et al., 2025). However, research findings from different investigators demonstrate variability in the extent of nutrient availability enhancement and pH alterations caused by these amendments. The differences in study results may be due to a variety of factors, such as climate, soil properties, fertilisers, and planted plants. Therefore, numerous factors must be considered when analysing changes in soil properties. The changes in alkali-hydrolysed nitrogen content and pH in the B treatment were more pronounced than those in the OF treatment, which may be related to the nature of the biochar. Biochar is a carbon-rich product of pyrolysis (Xu et al., 2025). It contains a substantial amount of stable carbon, which can increase soil carbon after being added to the soil, and the pH of biochar rises after the pyrolysis process due to the breakdown of alkali salts and functional groups (Abujabhah et al., 2016).

After the soil is applied, its pH value increases. Many studies have shown that biochar has a rich microporous structure and a large specific surface area, which makes it easy to adsorb small molecular organic matter in the soil and form macromolecular organic matter via polymerisation (Yang et al., 2023). The effect of OF on increasing available potassium and phosphorus is greater than that of biochar, possibly because the application of OF introduces a significant amount of activated carbon into the soil (Yang et al., 2023), which accelerates microbial reproduction and the bioactivation of potassium-bearing minerals, thereby increasing the amount of available potassium. In contrast, the increase in available phosphorus is primarily attributed to the decomposition of organic matter (Jindo et al., 2023; Zhang et al., 2022).

The organic matter content of the OF treatment was not significantly different from that of the control after 1 year of treatment, indicating that the mineralisation decomposition rate was high. Meanwhile, the B treatment remained significantly higher than the control, demonstrating that biochar had a strong advantage in improving SOM. The readily decomposable carbon components (e.g., free sugars) found in organic fertilisers, such as crop straw and manure, can be quickly utilised by microorganisms, resulting in an increased short-term mineralisation rate (Luo et al., 2019; Mo et al., 2022). This aligns with the dynamic pattern of organic matter increase in the early stage and decrease in the later stage of the OF treatment in this study. Biochar has a highly aromatic structure and exhibits strong resistance to microbial decomposition, allowing it to retain carbon for an extended period. Moreover, the pore structure of biochar may indirectly reduce losses by adsorbing soluble organic matter.

### 4.4 Effects of biochar and OF on soil bacterial diversity

Soil microecology is an important index of soil health. The composition and abundance of soil microorganisms reflect soil microecology to a certain extent (Jurys and Feiziene, 2021). In this paper, the amplicon sequencing data showed that the bacterial *α* diversity indices (such as Shannon and Chao1) for biochar and OF were lower than those for single application, which contradicts the findings of previous studies (Hu W. et al., 2024). Organic fertilisers may stimulate the explosive proliferation of certain eutrophic microorganisms (such as actinomycetes) and inhibit the niche of other oligotrophic microorganisms by rapidly releasing high concentrations of soluble nutrients (such as NH₄ and DOC) (Tao et al., 2020).

The high pore structure and adsorption characteristics of biochar may change the local microenvironment (such as pH and oxygen content) in the short term, resulting in a fluctuation in the abundance of some sensitive groups (Su et al., 2024). It should be noted that the correlation between localised shifts in sensitive taxa and holistic community diversity metrics requires further validation through long-term observational studies. Compared to CK, the combined application of biochar and organic fertiliser exhibited no significant effects on alpha diversity. This phenomenon may be attributed to the synergistic effects between organic fertiliser and biochar in counteracting environmental pressures; biochar effectively adsorbs excess nutrients, thereby alleviating the “eutrophication effect” induced by organic fertiliser (Tian et al., 2025). Concurrently, its porous structure provides physical refuge for microorganisms and facilitates differential colonisation of functional communities (Tian et al., 2025). This mechanism aligns with the “niche complementarity hypothesis” proposed by Zhou et al. (2022), which posits that multi-substrate inputs can enhance microhabitat heterogeneity, thereby mitigating the inhibitory effects associated with single-substrate applications. Previous studies have shown that bacteria can directly promote plant growth through their inherent biological activities and also mediate rhizosphere microflora remodelling by inducing the enrichment of other populations, collectively enhancing the growth-promoting ability of plants in rhizosphere ecosystems (Sun et al., 2022; Zhang et al., 2021).

The amendment of soil with biochar and organic fertilisers has been systematically investigated regarding their modulatory effects on microbial community structure and diversity. Comparative analyses have revealed differential impacts between these two soil amendments on microbial population dynamics. Notably, the introduction of exogenous carbon sources with distinct chemical properties induces substantial shifts in soil microbial community composition, potentially altering functional gene expression profiles and metabolic pathways within soil microbiomes (Wang et al., 2024). However, current research primarily focuses on cataloging microbial community responses to various carbon amendments, while critical mechanistic questions remain unresolved. A significant knowledge gap persists in understanding how these induced microbial community alterations translate into functional changes at the microbiome level and subsequently influence crop performance through plant-microbe-soil interactions. This functional elucidation requires integrated multi-omics approaches combined with targeted phenotypic analyses to establish causal relationships between microbiome restructuring and agricultural productivity outcomes. LEfSe analysis showed that there were few differentially expressed microorganisms in the control treatment, whereas the application of biochar and OF enriched a large number of microorganisms. Among them, *Sphingomonas* was significantly enriched in treatment B, indicating that *Sphingomonas* may play a key role in the growth of cut roses. Previous studies have found that *Sphingomonas* can decompose various organic compounds, help improve soil quality, and play an important role in maintaining plant growth and development (Wu et al., 2024), possibly because biochar is rich in stable carbon sources, such as carboxyl, hydroxyl, carbonyl, and aromatic hydrocarbons. These surface functional groups stimulate the growth of *Sphingomonas* (Yang et al., 2024).

In addition, the application of biochar significantly enriched microbial groups with specific ecological functions. At the order/family level, Rhizobiales (Rhizobiales) members such as Bradyrhizobium have been shown to promote symbiotic nitrogen fixation, while genera such as Arthrobacter in Micrococcales (Micrococcales) show strong organic matter degradation ability. At the class level, the microorganisms of the Sphingomonadaceae family contained in Alphaproteobacteria have the characteristics of pollutant degradation (He et al., 2022). These groups are involved in the processes of nitrogen and phosphorus transformation (Rhizobiales), lignin decomposition (Micrococcales), and pathogen antagonism (Streptomyces genus of Actinobacteria) through synergistic effects, and the optimisation of their community structure directly improves soil fertility and crop disease resistance (Ding et al., 2023; Dong et al., 2024) and is important for improving crop productivity. In OF treatment, *Gammaproteobacteria*, *Xanthomonadales* and *Rhodanobacteraceae* were enriched. *Gammaproteobacteria* can widely colonise the endophytic layer and rhizosphere of plants and have the potential to promote plant growth and regulate plant health and metabolism (Esma et al., 2020). *Xanthomonadales* can reduce pathogens, improve soil conditions, and promote plant growth. *Rhodanobacteraceae* have also been reported to be significantly enriched after straw returning (Sun et al., 2021) and may play an important role in plant growth promotion, disease prevention, and control. This result could be attributed to the fact that OF is rich in a large amount of active organic carbon, which can significantly increase the abundance of beneficial microorganisms, thus affecting the rhizosphere soil microecology of plants and improving crop productivity. Rare microbial groups play important roles in maintaining the ecosystem, microbial community structure, and biogeochemical cycle (Chen et al., 2020). Therefore, the introduction of biochar and OF, through their carbon structural characteristics, significantly altered the SOC composition and induced transformations in bacterial ecophysiological strategies, fostering more active and co-cultured bacterial communities. This model may closely relate to the response of plant productivity to soil multifunctionality.

In this study, the combined application of biochar and OF not only recruited beneficial microorganisms but also increased the abundance of certain microorganisms, such as Chloroflexi and *Burkholderiales*. Chloroflexi can participate in biogeochemical cycles, such as carbon, nitrogen, and sulphur; promote the stability of microbial community structure and the removal of nutrients (Xian et al., 2020); and *Burkholderiales* utilise both recalcitrant and degradable organic carbon (Cong et al., 2018). They can produce a variety of metabolites with antibacterial activity, such as siderophores and monoterpene alkaloids, playing an important role in maintaining community stability and supporting plant growth (Fan et al., 2021). The rare groups in soil treated with biochar and OF may play a more important role in sustaining plant productivity than those in soil treated with chemical fertiliser alone. Therefore, the contribution of rare but highly active microorganisms to plant productivity may surpass expectations based on their abundance.

Through correlation analysis and redundancy analysis, we found that soil carbon fractions (FOC and SOM) and physicochemical properties (AP) collectively explained 80.14% of the genus-level variation in the bacterial community, with the RDA’s first constraint axis contribution rate at 71.38%. Among these, FOC (soluble organic carbon), AP (available phosphorus), and SOM (organic matter) were identified as core factors driving microbial community reconstruction (*p* < 0.01). Biochar and organic fertiliser affect microbial communities through distinct carbon source characteristics. The former specifically stimulates the proliferation of specific microorganisms, such as Chloroflexi, through aromatic hydrocarbons, while the latter significantly increases the abundance of beneficial bacteria, such as Pseudomonadaceae, through active organic carbon, including low molecular weight organic acids. Notably, the combined application of both showed a synergistic effect: it not only enhanced the colonisation of growth-promoting bacteria such as Bacillaceae (*p* < 0.05) but also significantly increased the alpha diversity of groups, with the Shannon index rising by 38.7%. This microbial network reconstruction may promote plant productivity by enhancing nutrient mineralisation and hormone secretion pathways.

Based on the above findings, we propose three directions for future research: (1) using ^13^C isotope tracing technology to quantitatively analyse the distribution flux of exogenous carbon in the chain of organic fertiliser-biochar-microbial metabolism-plant absorption; (2) establishing a microbial functional gene database to reveal the regulatory mechanisms of carbon source-induced community changes on greenhouse gas emissions; and (3) developing “synthetic microbiota” based on microbial functional modules to evaluate their ecological resilience under extreme precipitation and drought scenarios. These studies will promote the transformation of microbiome engineering from descriptive research to predictive regulation and provide a new paradigm for addressing the problem of soil degradation in the context of global warming.

## 5 Conclusion

This study provides evidence that the application of biochar and organic fertiliser has different effects on soil physicochemical properties and microbial community structure. In the intensive production of cut roses, the addition of organic carbon sources enhances productivity by regulating soil function and microbial community composition. Different carbon sources influence soil function through distinct pathways: organic fertilisers, which contain highly active carbon, promote the proliferation of beneficial microorganisms and enhance the availability of potassium (K) and phosphorus (P). In contrast, biochar, characterised by its highly stable carbon content, improves the quality of organic matter and nitrogen conversion efficiency by stimulating specific functional microbial communities, such as *Sphingomonas*.

The synergistic effects of carbon source application arise from their ability to activate specific microbial groups. This study elucidates, for the first time, the differential mechanisms by which exogenous carbon sources regulate soil microbiota in facility flower cultivation, providing a theoretical foundation for the synergistic effects of ‘carbon input, microbiome optimisation, and soil function improvement’ through precise carbon source management. These findings pave the way for new avenues for sustainable agricultural development.

## Data Availability

The original contributions presented in the study are included in the article/Supplementary material, further inquiries can be directed to the corresponding author.

## Footnotes

^1^ http://ccb.jhu.edu/software/FLASH/

^2^ https://www.r-project.org/
