# Supplementary material for: Different active exogenous carbons improve the yield and quality of roses by shaping different bacterial communities
Source: Front Microbiol. 2025 Mar 28;16:1558322. doi: 10.3389/fmicb.2025.1558322 (PMC11985833; doi:10.3389/fmicb.2025.1558322)
Supplement: Supplementary file 1 [file Data_Sheet_1.zip › Fig.4c.pdf]

Cladogram

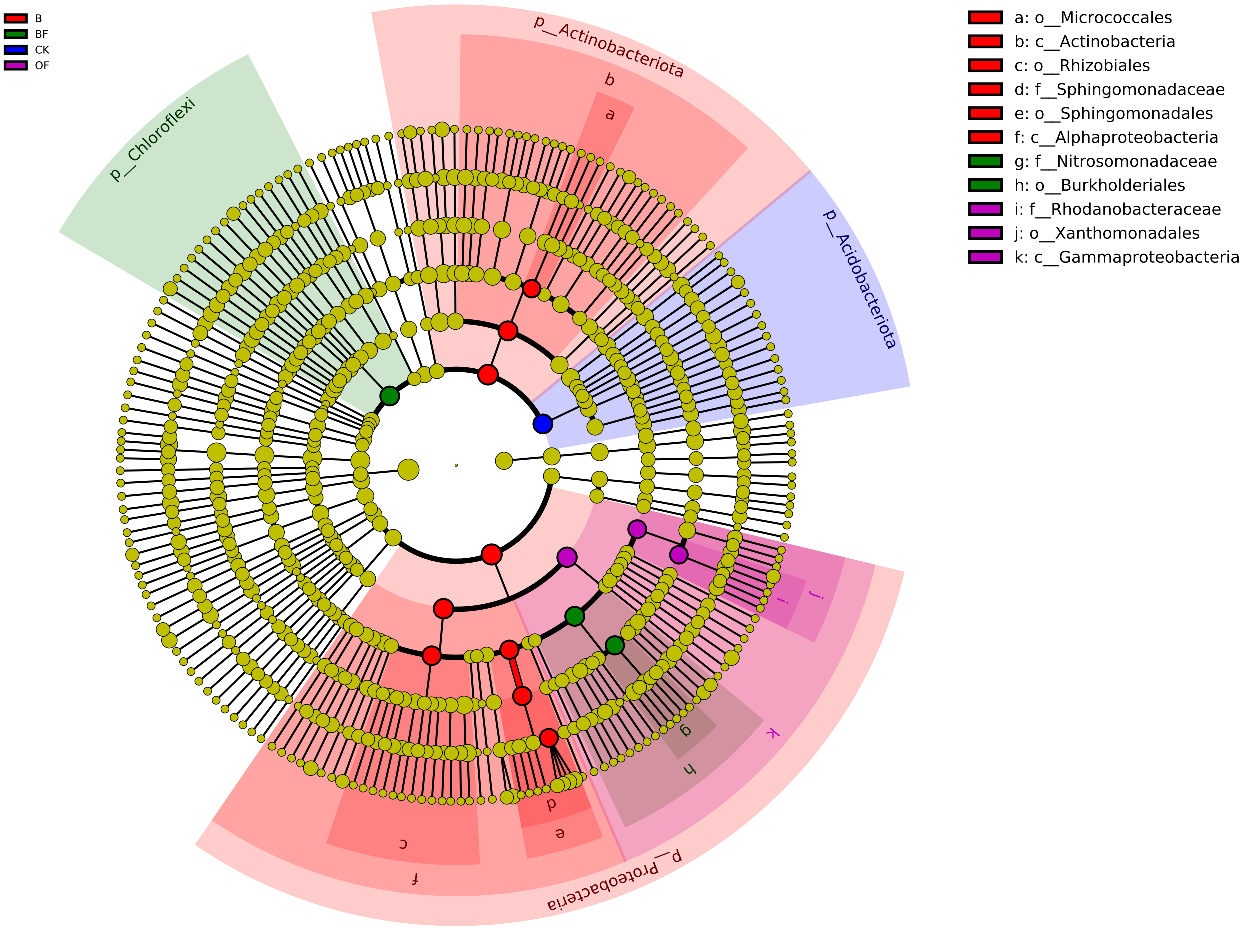

B
BF
CK
OF
p__Actinobacteriota
p__Chloroflexi
p__Acidobacteriota
p__Proteobacteria
a
b
c
d
e
f
g
h
i
j
k
a: o__Micrococcales
b: c__Actinobacteria
c: o__Rhizobiales
d: f__Sphingomonadaceae
e: o__Sphingomonadales
f: c__Alphaproteobacteria
g: f__Nitrosomonadaceae
h: o__Burkholderiales
i: f__Rhodanobacteraceae
j: o__Xanthomonadales
k: c__Gammaproteobacteria
