# Supplementary figures and images for: Different active exogenous carbons improve the yield and quality of roses by shaping different bacterial communities
Source: Front Microbiol. 2025 Mar 28;16:1558322. doi: 10.3389/fmicb.2025.1558322 (PMC11985833; doi:10.3389/fmicb.2025.1558322)

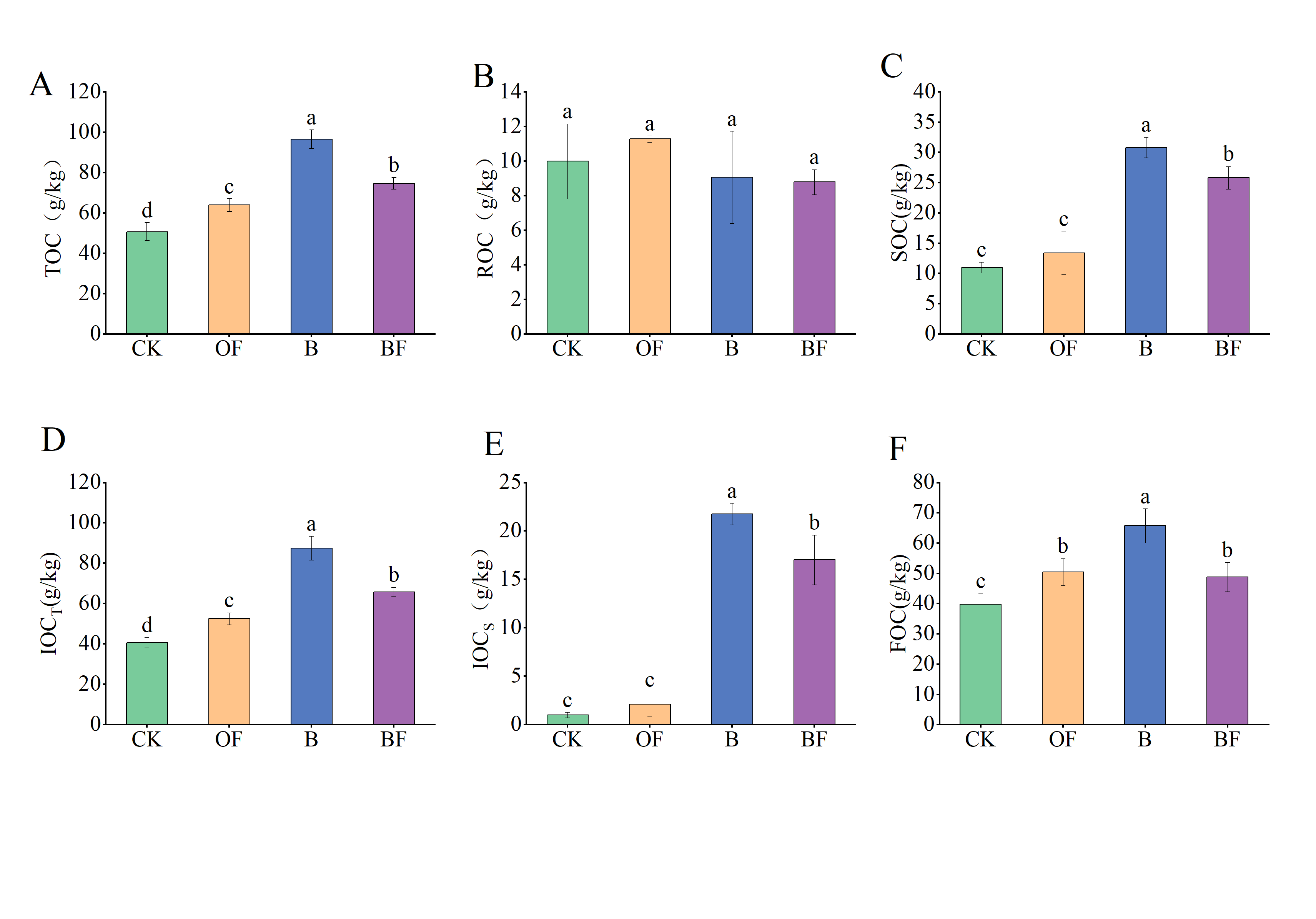

Supplement: Supplementary file 1 [file Data_Sheet_1.zip › ║╧▓ó╠╝╫Θ╖╓═╝.tif]

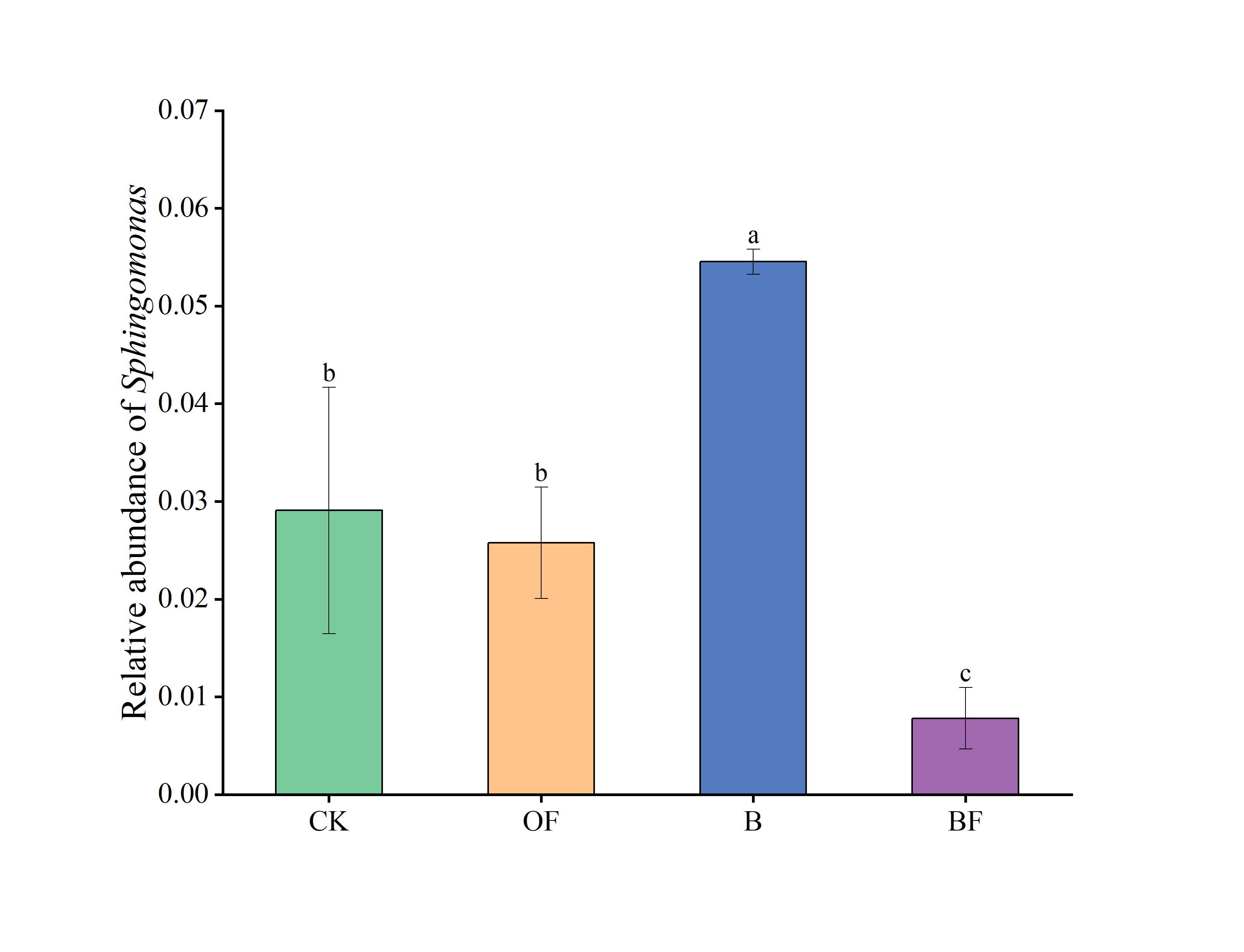

Supplement: Supplementary file 1 [file Data_Sheet_1.zip › Relative abundance of Sphingomonas.tif]

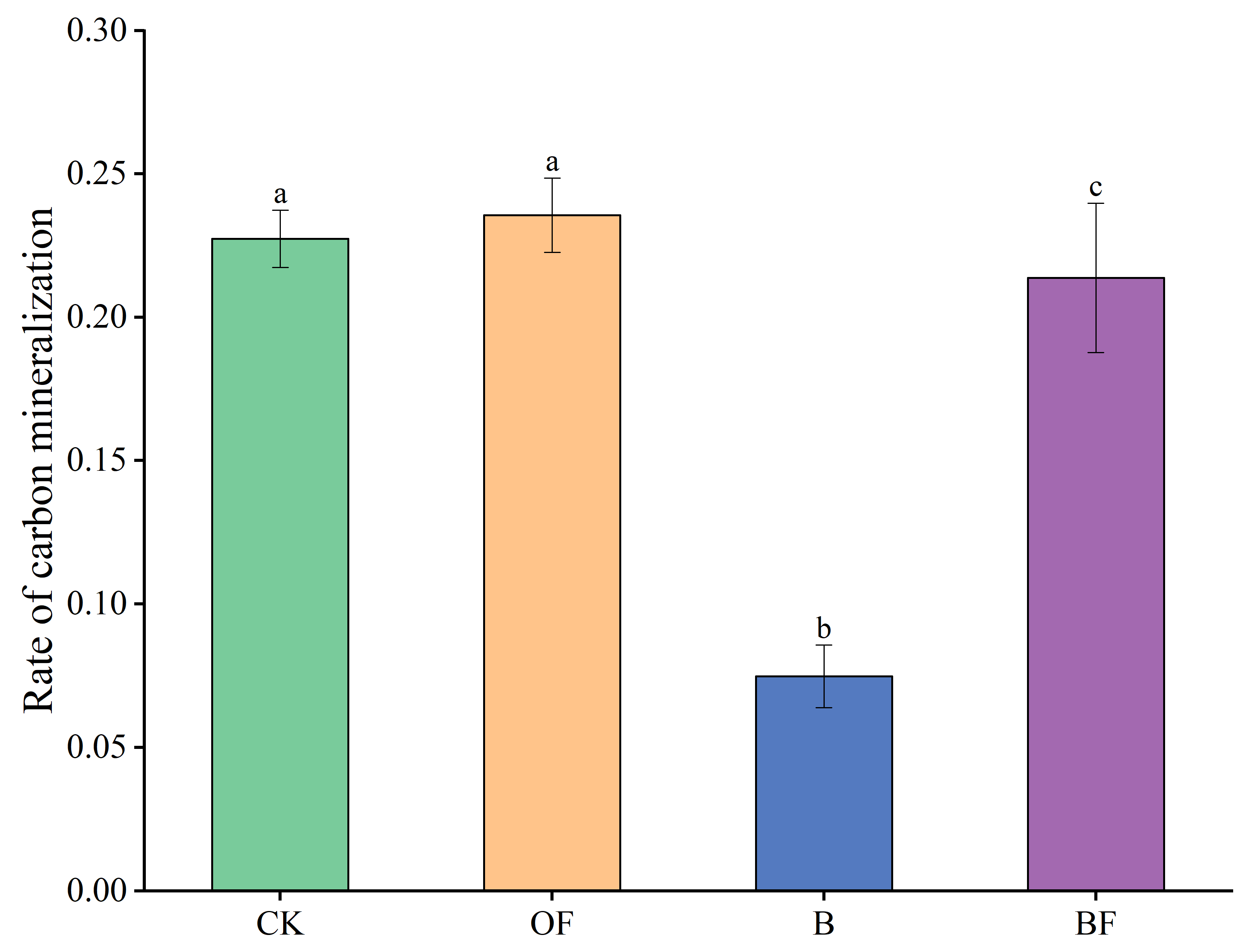

Supplement: Supplementary file 1 [file Data_Sheet_1.zip › Rate of carbon mineralization.tif]

# Correlation heatmap

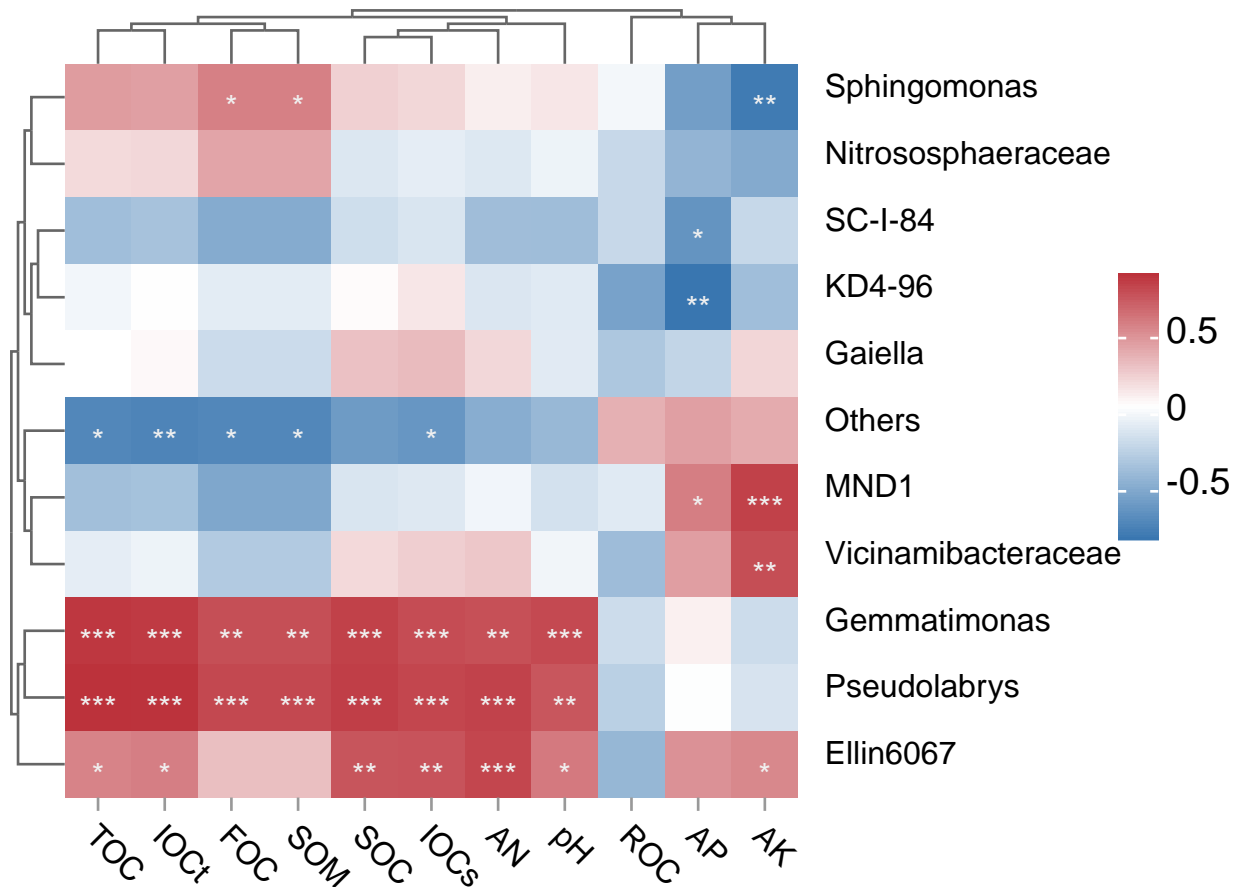

Supplement: Supplementary file 1 [file Data_Sheet_1.zip › Fig.5a.pdf]

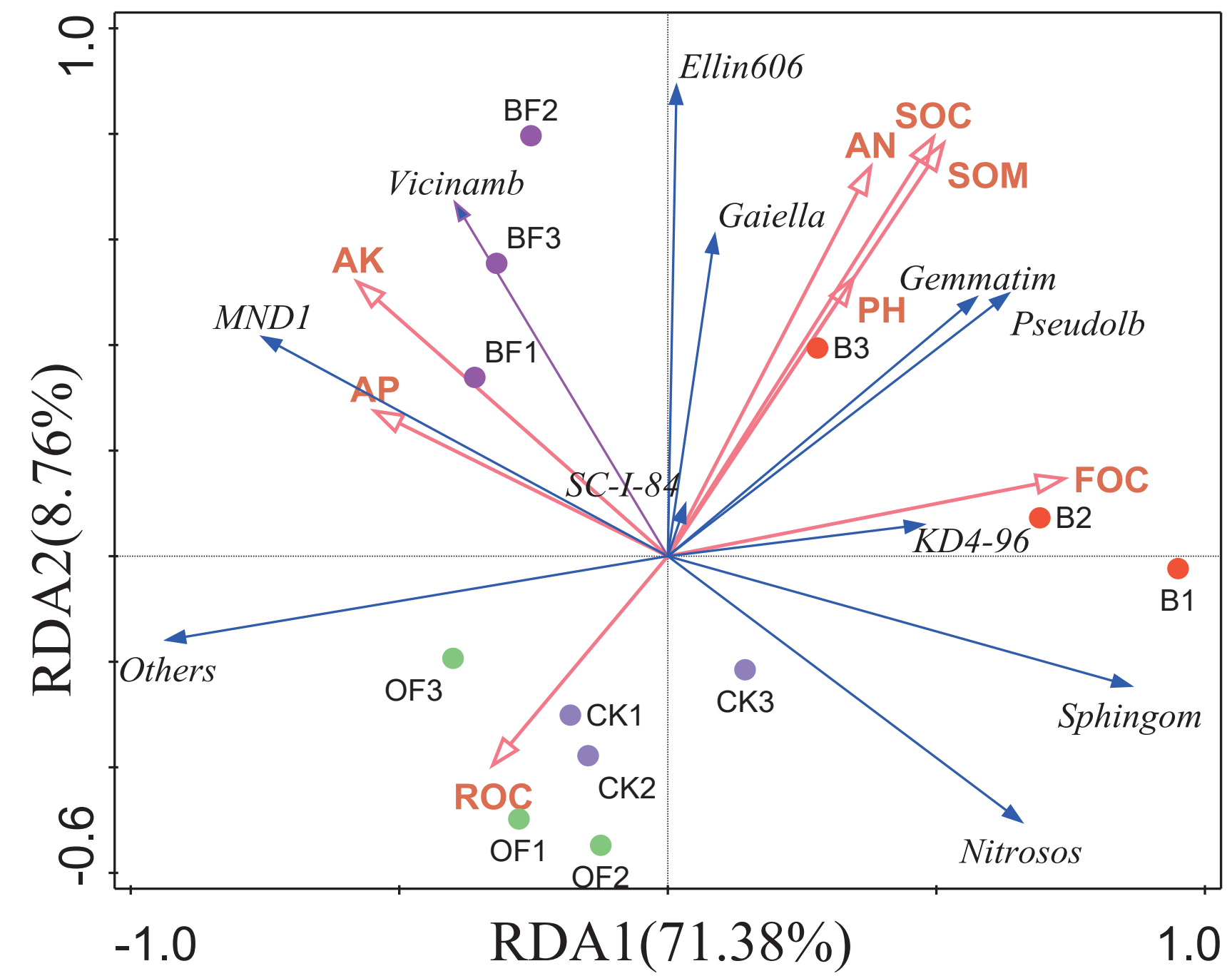

Supplement: Supplementary file 1 [file Data_Sheet_1.zip › Fig.5b.pdf]

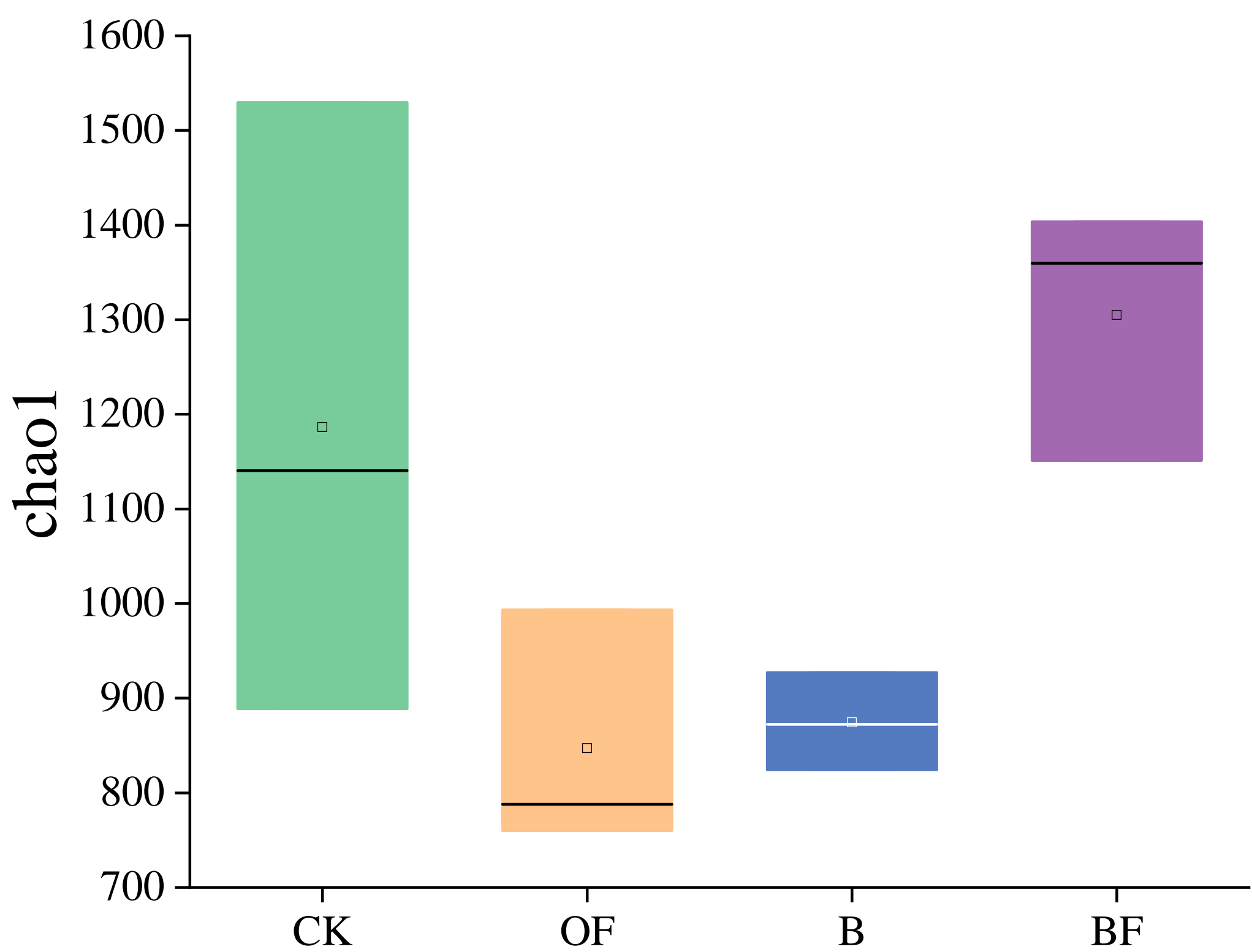

Supplement: Supplementary file 1 [file Data_Sheet_1.zip › Fig.3a.pdf]

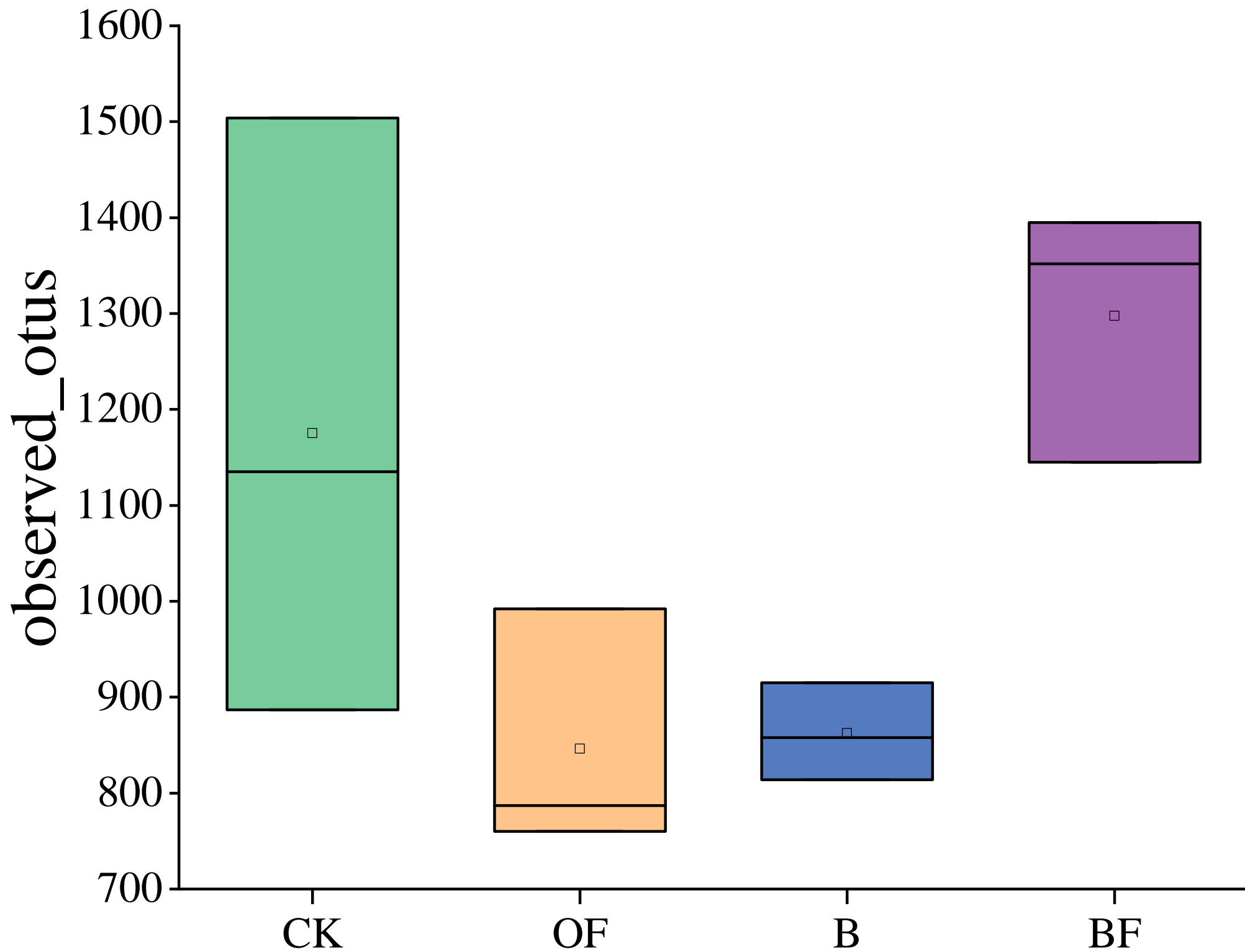

Supplement: Supplementary file 1 [file Data_Sheet_1.zip › Fig.3b.pdf]

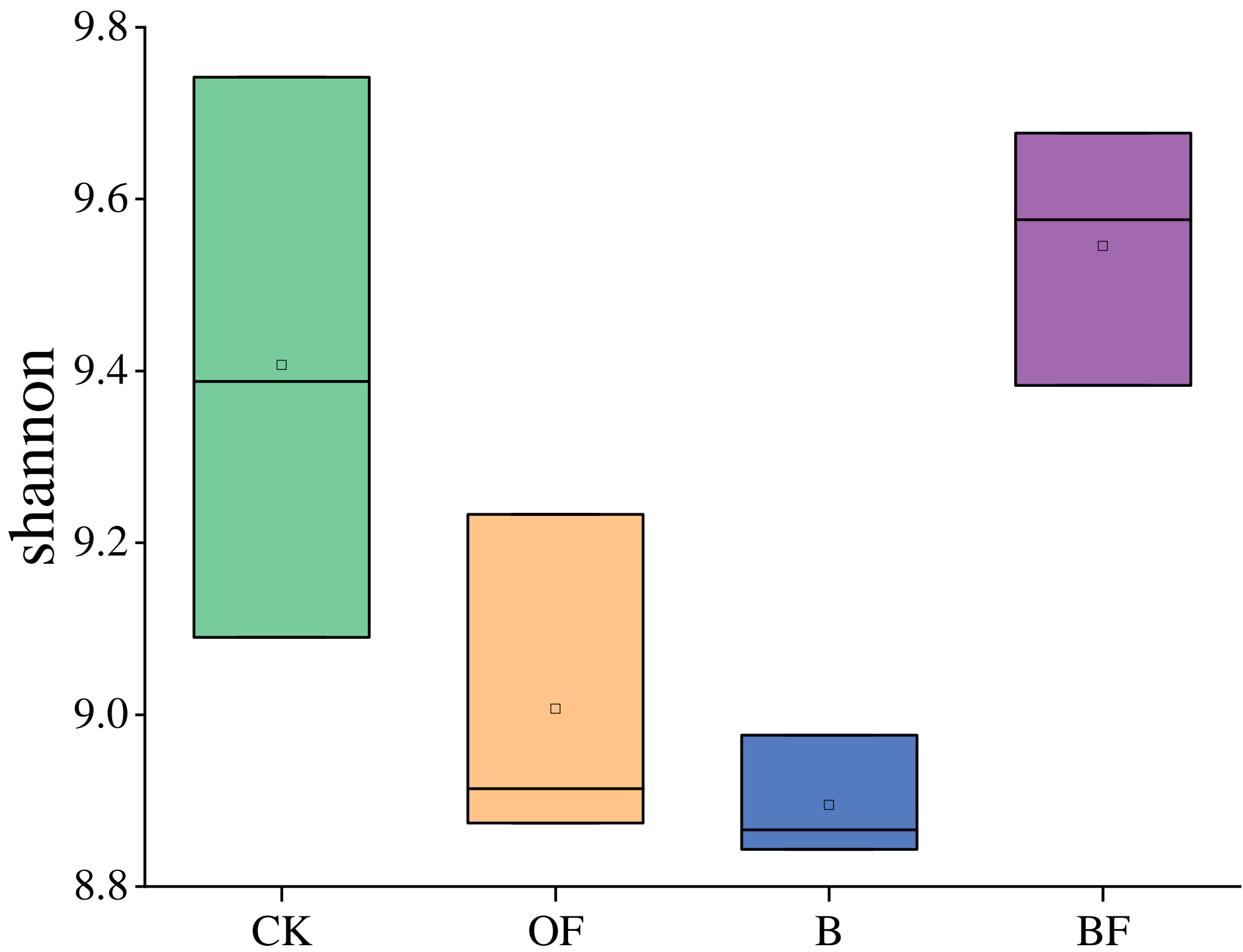

Supplement: Supplementary file 1 [file Data_Sheet_1.zip › Fig.3c.pdf]

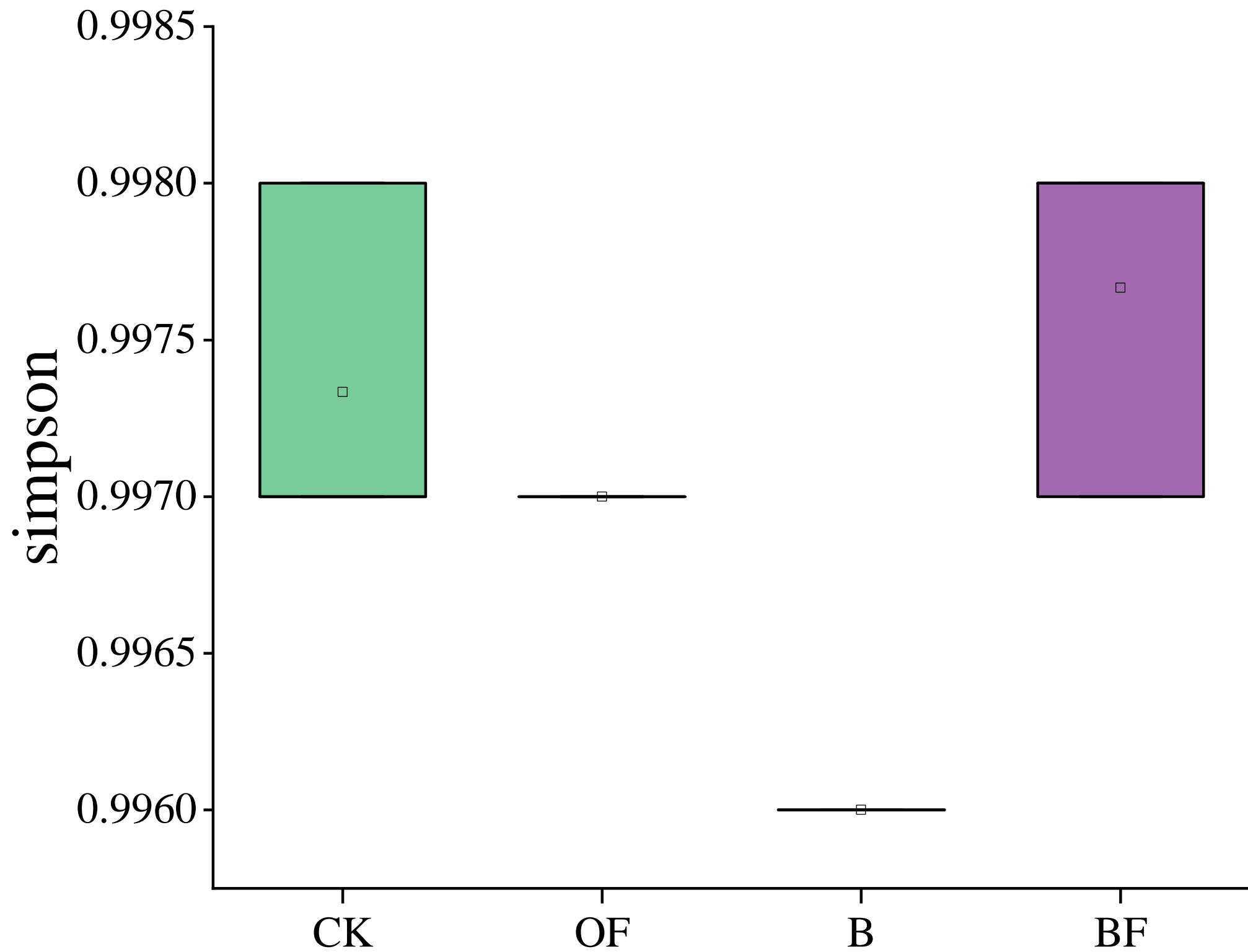

Supplement: Supplementary file 1 [file Data_Sheet_1.zip › Fig.3d.pdf]

NMDS Plot

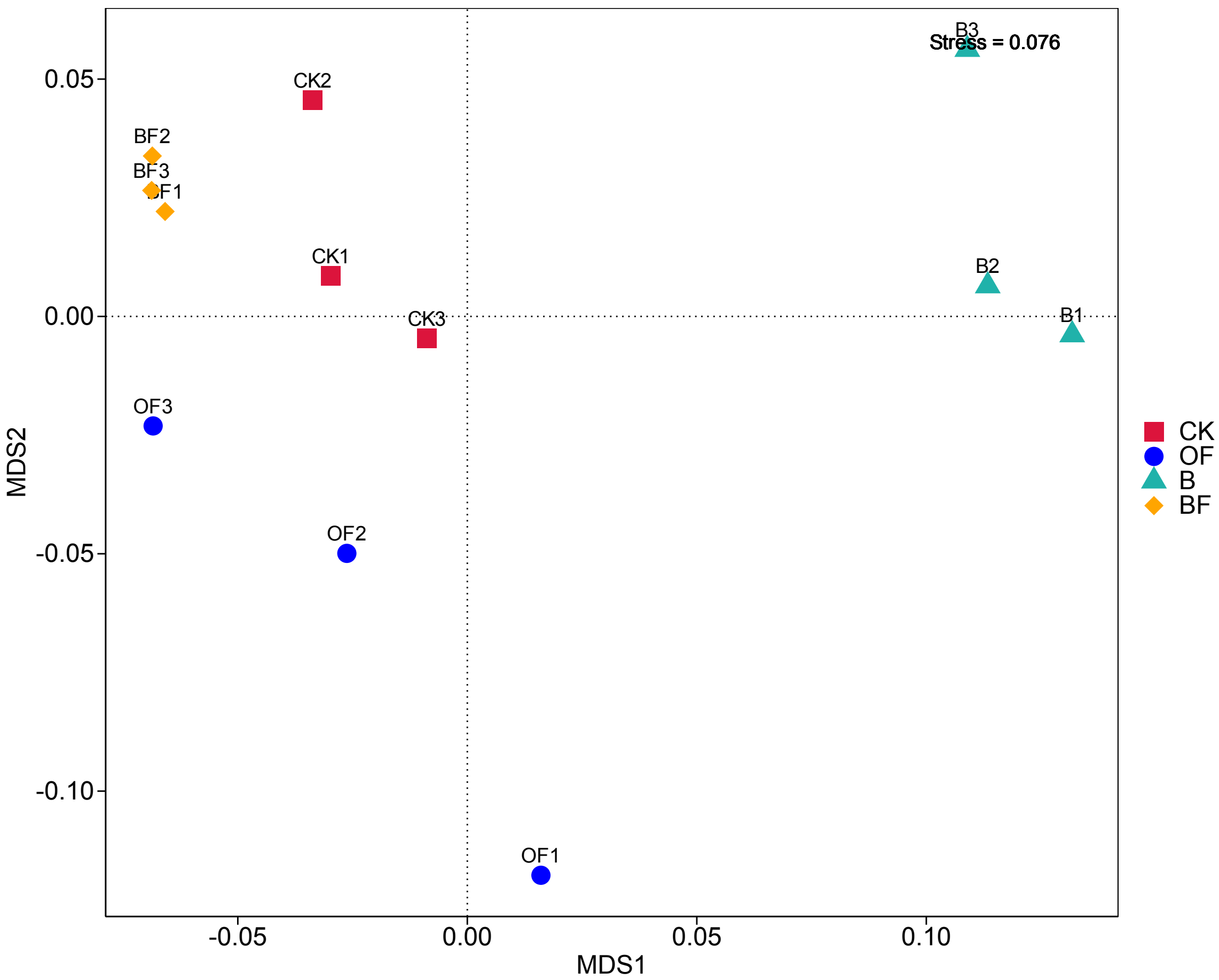

Supplement: Supplementary file 1 [file Data_Sheet_1.zip › Fig.4a.pdf]

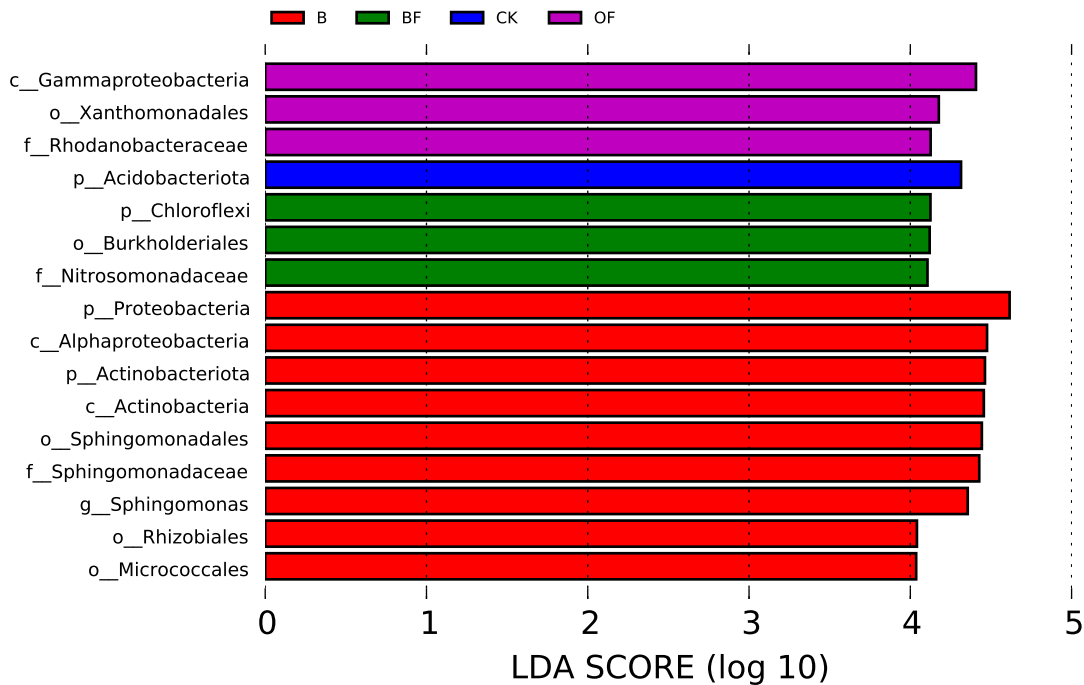

Supplement: Supplementary file 1 [file Data_Sheet_1.zip › Fig.4b.pdf]
